# Comparisons of weed community, soil health and economic performance between wheat-maize and garlic-soybean rotation systems under different weed managements

**DOI:** 10.7717/peerj.4799

**Published:** 2018-05-30

**Authors:** Mahmud A. Muminov, Liyue Guo, Yanjie Song, Xian Gu, Yu Cen, Jie Meng, Gaoming Jiang

**Affiliations:** 1 State Key Laboratory of Vegetation and Environment Change, Institute of Botany, Chinese Academy of Sciences, Beijing, China; 2 University of Chinese Academy of Sciences, Beijing, China; 3 Laboratories of Environmental Problems, Samarkand State University, Samarkand, Uzbekistan

**Keywords:** Weed community, Crop rotation, Wheat, Soybean, Weed management, Soil health, Earthworms, Eco-economic benefits

## Abstract

This study compared the impacts of different weed managements on weed community, soil health and economic performance between the wheat–maize (WM) and garlic–soybean (GS) rotations. A total of four treatments (H_0_T, tillage without herbicide; H_0_T_0_, without both herbicide and tillage; HT, both herbicide and tillage; HT_0_, herbicide without tillage) were designed for both rotations. A total of 16 weed species were recorded in the WM rotation, with life forms of 62% for annuals, 12% for annual + perennial and 20% for perennials. While in the GS rotation, there were 17 weed species, with 71% being annuals. When crop rotation changed from WM to GS, the topsoil layer seed bank (0–5 cm) decreased by 137%. GS rotation always had higher earthworm densities than that of WM under the same condition. Organic weed control (H_0_T, H_0_T_0_) from both WM and GS added more soil organic matters than the chemical methods (HT and HT_0_). Economically, up to 69% higher net profit had been achieved in the GS than WM for their organic products. This study provides an ecological basis to guide organic farming practices, especially for weed management in the future.

## Introduction

Globally, competition from weeds has led to crop yield losses by as large as 40% when all major crops are averaged (Oerke, 2006). Over the past 40 years, traditional weed controlling methods such as human, animal, and mechanical weed control have largely been replaced by herbicides, and they have made a significant contribution to the high productivity of global agriculture (Powles & Yu, 2010). However, it was found that weed species have never been eradicated by the use of herbicides, on the contrary, a series of new problems have occurred, such as environmental pollution, decrease of biodiversity, degradation of food quality, and human health problems (Altieri & Hawksworth, 1991; Ju et al., 2009; Guo et al., 2015).

Scientists, government officials, and farmers have made great efforts in facing the challenges posed by weeds. Unfortunately, their reliance on innovated herbicides has led to the increase in the amount of herbicide applications. Since the 1980’s, for instance, China’s consumption of herbicide had increased from 23,000 tons to more than 3.4 million tons in 2010 (National Bureau of Statistics of China, 2011). The increasing application of herbicide is accompanied by the evolution of herbicide resistance in weeds, and weed species population shifts. Meanwhile, herbicide resistant in weeds caused serious problems for those new developed crops with herbicide tolerance (Bajwa, 2014). Therefore, a wise understanding of weed biology and ecology may lead to effective use of herbicides through ideal cultivation practices. From this point of view, some sustainable and environmentally friendly weed control theories and techniques are urgently needed.

Crop rotation and tillage have been considered crucial approaches to reduce herbicide pollution and maintain yield through building up an integrated weed management system (Swanton & Murphy, 1996). Crop sequence diversification has effects on soil seed bank, due to an alternate habitat that weed cannot successively adapt to or expand. For example, differences in weed populations have been observed between monoculture maize and a rotation of maize and soybean, the latter being phenologically different crop. Crop rotation associated with legume species can also increase soil fertility (Doucet et al., 1999; Kegode, Forcella & Clay, 1999). Therefore, crop rotation is believed to be an ideal method for weed control (Liebman & Dyck, 1993; Bachinger & Zander, 2001, 2007). Conservation tillage may strongly affect the germination of weed seeds by increasing top soil layer moisture and temperature (Froud-Williams, 1988). While after rotary tillage, many weed species depending on a single regeneration strategy can be controlled as most of the seeds are buried by moldboard plowing (Ball, 1992; Buhler, 1992). Crop rotation and tillage also influence the soil environment through inputs and disturbance of the soil, which in turn, impact soil quality (Zuber et al., 2017).

Any integrated weed management plan or strategy should link the most economical and effective means of weed control with ecological considerations. The information on weed flora dynamics, weed biology, and ecology is believed to be very important to beneficially approach environmental and economic weeding strategies (Albrecht, 2005). Therefore, the effects of crop rotation, tillage and herbicide on weed population dynamics, soil health, economic, and environmental performance should be carefully examined.

Organic agriculture is characterized by the prohibition of synthetic chemicals in both crop production and livestock raising (Lampkin, 2002). We hypothesized that organic products yielded higher income and weeds can be controlled following the suitable crop rotations and tillage. In this study, winter wheat (*Triticum aestivum*)—summer maize (*Zea mays*) rotation and soybean (*Glycine max*)—garlic (*Allium sativum*) rotations were selected in North of China to focus on two key points: (1) the effects of no-herbicides and herbicides weed management on floristic composition and weed communities in different crop rotation systems; (2) the influences of crop rotation on soil seed bank and their economic performances. Some of the new findings from this study might help to solve herbicide pollution and increase both soil and food quality.

## Methods

### Study site

**Figure 1:**
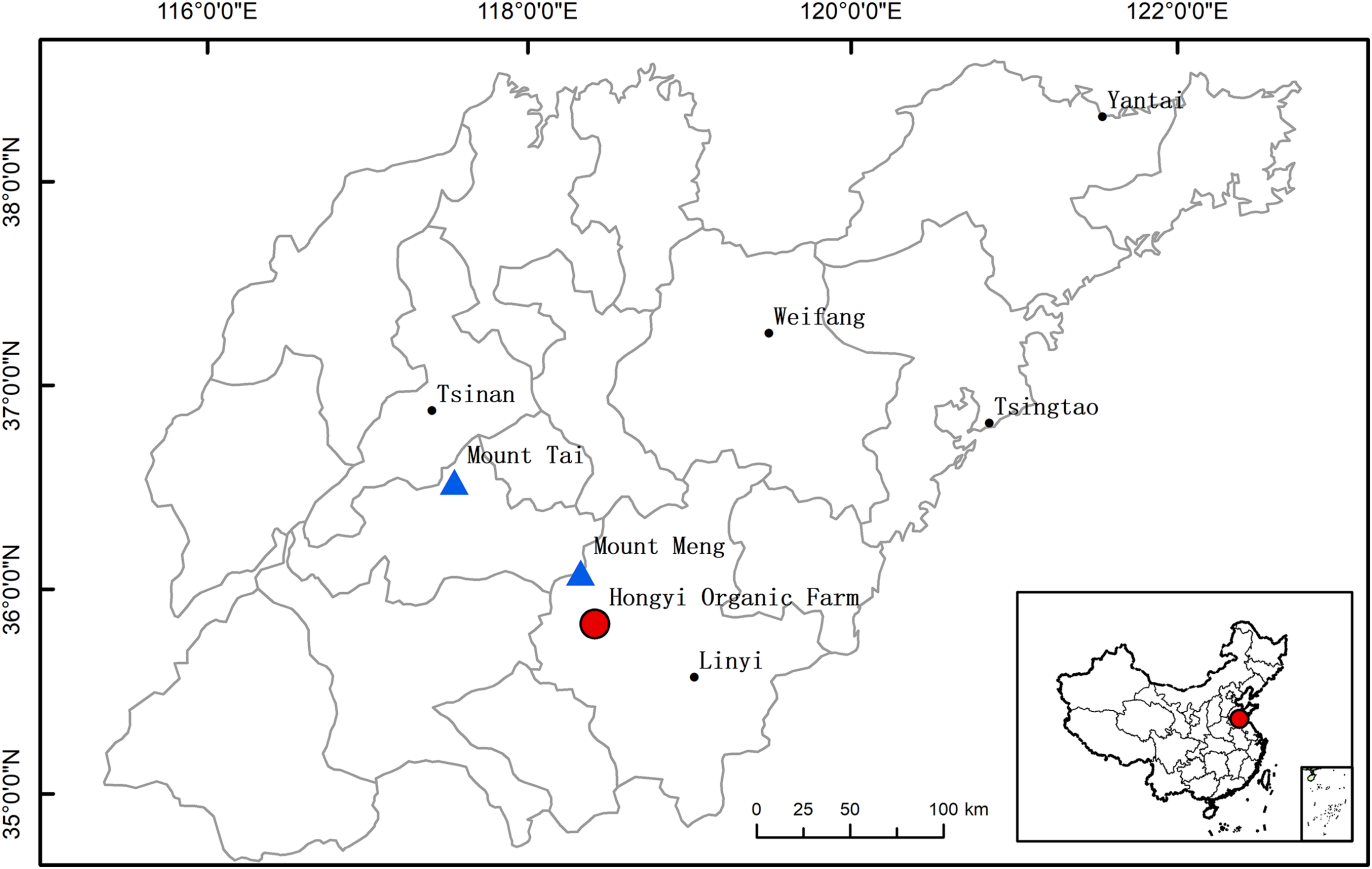
The location of Hongyi organic farm.

**Figure 2:**
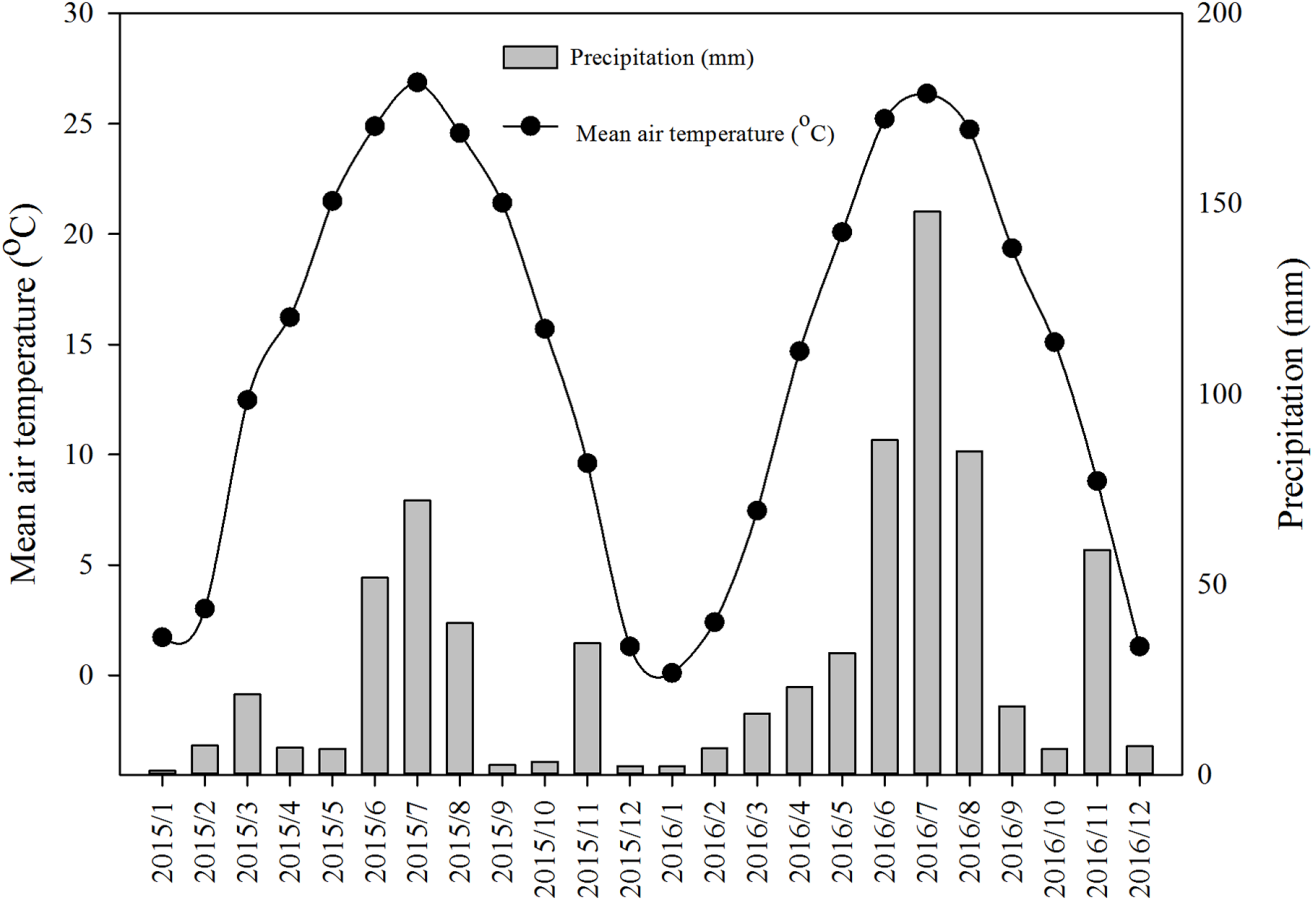
Monthly mean air temperature and precipitation during 2015–2016 in the study area.

The field experiment was conducted in Hongyi Organic Farm, Pingyi County, Shandong Province, China (35°26′21″N, 117°50′11″E), which was approved by the Plant Eco-physiological Research Group in the Institute of Botany, Chinese Academy of Sciences (project number: 201405.5). The location of study area is shown in Fig. 1. The climate of the study area is characterized as typical temperate and monsoonal, with mean annual precipitation and temperature being 770.2 mm and 13.2 °C respectively (Fig. 2). The soil type is yellow–brown. Before experiment, the soil organic matter (SOM) was measured to be 1.22%, total nitrogen 0.09%, soil bulk density 1.48 g · cm^−3^, soil earthworm density and biomass 63 No. · m^−2^ and 16.4 g · m^−2^, respectively. Conventional crop rotation in the study area is winter wheat and summer maize. Soybean, peanut (*Arachis hypogaea*), and garlic are seldom planted. Rotation of winter wheat-summer maize and soybean–garlic were investigated here. Winter crops (wheat and garlic) grow from early October to early June of the following year; while summer ones (maize and soybean) grow from middle June to early October of the same year.

### Experimental design

The experiment started from June 2014 and stopped in October 2016. A total of four treatments (H_0_T, tillage without herbicide; H_0_T_0_, without both herbicide and tillage; HT, both herbicide and tillage; HT_0_, herbicide without tillage) were designed for both wheat–maize (WM) and garlic–soybean (GS) rotation (Table 1). Tillage (T) was done before sowing (primary plowing with a depth of 20–25 cm). Weed was controlled by either herbicide (H) or manual weeding (H_0_). Each treatment (plot size 2.4 × 16 m) was arranged with three replicates. Cattle manure compost contained organic matter 42.4%, total nitrogen 1.7%; available phosphorus 0.25% and available potassium 0.56% was applied at the rate of 7.5 tons · ha^−1^ (wet weight) only before sowing winter crops. Wheat was seeded by a machine under 10 rows per plot with a row width of 20 cm. Garlic was sowed by hand along with rows being 20 cm and distances being 10 cm between plants. Summer crops were seeded in June 15 (for maize) and 20 (for soybean). Maize was seeded by a machine with a row width being 60 cm and distances between plants being 10 cm. Soybean was manually sowed with a row width being 40 cm and distance being 25 cm. The varieties of maize, soybean, wheat, and garlic were Zhengdan 958, Xudou 14, Liangxing 99, and Zhongsuan 1, respectively.

**Table 1:** Treatment designed in summer and winter crop rotation system.

Treatments	Rotation	From Oct., 2014 to June, 2015	From June to Oct., 2015	From Oct., 2015 to June, 2016	From June to Oct., 2016
**H_0_T**	WM	Wheat	Maize	Wheat	Maize
	GS	Garlic	Soybean	Garlic	Soybean
**H_0_T_0_**	WM	Wheat	Maize	Wheat	Maize
	GS	Garlic	Soybean	Garlic	Soybean
**HT**	WM	Wheat	Maize	Wheat	Maize
	GS	Garlic	Soybean	Garlic	Soybean
**HT_0_**	WM	Wheat	Maize	Wheat	Maize
	GS	Garlic	Soybean	Garlic	Soybean

**Note:**

H_0_T, tillage without herbicide; H_0_T_0_, without herbicide and tillage; HT, both herbicide and tillage; HT_0_, herbicide without tillage; WM, winter wheat and summer maize rotation; GS, garlic and soybean rotation.

### Weed biomass and density

Weed data was collected before each weeding time at each crop season. Assessment of weed communities was conducted randomly in three replicates by 1 × 1 m size quadrates in each treatment. Weed species, number of the plant for each weed and total biomass of each sample plot were measured. Weed samplings were done two times at each crop season, 42 and 65 days after sowing for summer crops, 167 and 215 days after sowing for winter crops. Weed indicators were averaged and combined accordingly two crop rotation system (GS and WM). Weed summed dominance ratio (SDR, %) was calculated according to the weed density and biomass in different weed control treatments. The SDR was calculated as follows:

$${\rm{SDR}} = {{{\rm{RD}} + {\rm{RDW}}} \over 2} \times 100 \text {%} $$

RD, relative density, % (weed species density divided by total weed density) and RDW, relative dry-weight biomass, % (weed species dry-weight biomass divided by total weed dry-weight biomass) of each species in each plot (Bhagat et al., 1999). Two sampling times were averaged in each crop field.

### Soil organic matter

At the beginning and end of the experiment, soils were randomly sampled at the layers (0–20 and 20–40 cm) using a 5 cm inside diameter corer. Five subsamples were randomly taken in each plot, which was mixed together into one soil sample. The soil samples were air-dried and passed through a 100 mesh sieve for analyzing SOM. SOM was measured by potassium dichromate oxidation-ferrous sulfate titrimetry method (Nelson & Sommers, 1996).

### Soil bulk density, water content

Soil bulk density and water content were done at a soil depth of 0–20 cm. Bulk density was determined on undisturbed soil samples using a steel cylinder of 100 cm^3^ volume (5 cm in diameter, and 5.1 cm in height) which was calculated by dividing the weight of the dried soil by the volume of the soil (Black & Hartge, 1986). Soil water content was determined gravimetrically after drying at 105 °C for 24 h. All the above indicators were determined with three replicates in each treatment.

### Soil seed bank

Soil seed bank was investigated prior to the initiation of this study and was done in the middle of March 2017, following greenhouse germination methods (Cardina, Herms & Doohan, 2002). The determination was conducted after wheat and garlic planting, with 10 soil cores (5 cm in diameter and 20 cm deep) being obtained at random from each plot. An additional set of 10 soil cores obtained from each plot were divided into 0–5 and 5–20 cm depths level and all the cores of a given depth were pooled for each plot. The soils were sieved through a 0.64 cm screen to break up large soil samples.

Soil samples were washed through sieves with 4 and 0.25 mm screens for the separation of weed seeds. The retained contents were air-dried, sorted under an illuminated magnifier, with the seeds being identified and removed. The samples were placed on a sand bed and spread in a 22 cm^2^ tray and watered every day so that the soil surface was kept moist in the greenhouse. The greenhouse temperatures were set at 18 and 8 °C, for day and night, respectively, without artificial lighting. Emerged weed seedlings were identified, counted and removed. When emergence ceased, samples were stirred, re-sieved and placed in a 4 °C cold room for three weeks followed by one week at an alternating temperatures (15 and 4 °C, day and night) before being returned to the greenhouse. This process was repeated twice till no additional seedlings emerged. Data were generated by counting numbers of germinated seeds per square meter.

### Soil earthworm

Earthworms in the soil were investigated through hand-sorting method trimonthly in each experimental year. Three soil blocks were investigated in each plot at one sampling time. The size of the soil blocks was 30 cm (length) × 30 cm (width) × 20 cm (depth). The earthworms collected were brought to the laboratory for identification and counting. All determinations were done with three replications.

### Aboveground biomass and yield

At the harvest stage, aboveground biomass was determined in 2.4 × 1 m plot per treatment in three replications. The whole plants in each plot were transported to the laboratory for segmentation and then dried at 105 °C for 30 min and at 75 °C until reaching a constant weight. Crop yields were determined in another 2.4 × 1 m plot per treatment in three replications and weighed on air-dried condition. All the data were averaged for future analysis.

### Leaf area index and plant height

The leaf area index (LAI) was conducted using a SunScan canopy analyzer (Delta-T Devices, Cambridge, UK) (Potter, Wood & Nicholl, 1996). Crop height was conducted by manually using yard-measure. A total of 10 representative plants at the middle of row were measured from bottom to top of the plant and averaged. Such measurements were conducted at harvesting stage.

### Economic assessment

Comparisons of economic benefits were conducted between WM and GS rotation under different weed managements. We conducted economic analysis using online market organic price and conventional price, respectively for organic and non-organic productions. The incomes were calculated from both rotations under different treatments based on the different price levels and crop yields. Detailed data for the calculation for each category were presented in supplementary Table S1.

### Statistical analysis

Statistical data were analyzed by three-way ANOVA test to compare the significant effects (*P* < 0.05, *P* < 0.01, or *P* < 0.001) of rotation, tillage, and herbicide factors and four-way ANOVA test for rotation, tillage, year, and herbicide factors using the software SPSS 17.0 (SPSS, Chicago, IL, USA). To compare means, separations were conducted using multivariate Duncan test. Figures were generated using Sigma Plot 10.0 (Aspire Software Intl., Ashburn, VA, USA). All data of results were presented as means and standard error.

## Results

### Weed diversity and dominant species

A total of 16 weed species were recorded in the WM rotation system. Main weed species included *Portulaca oleracea*, *Acalypha australis*, *Echinochloa crus-galli*, *Humulus scandens*, *Calystegia hederacea*, *Cirsium setosum*, etc., in the maize field, and *Descurainia sophia*, *Capsella bursa-pastoris* and *C. setosum* in the wheat field. However, when the rotation changed to GS, the total number of weed increased to 17 species. Although the species number did not display a big change, the dominant species have changed. The life forms of the majority of weeds were annuals, with a few being perennials. In the WM rotation, 62% of the life forms were annuals, 12% annual + perennial and 20% perennials. While in the GS rotation, the ratio of annuals to total weed species increased to 71% (Table 2).

**Table 2:** Influence of the different weed management on summed dominance ratio (%) of major weeds during the 2015–2016 period.

Scientific name	Plant type	Maize field				Soybean field				Wheat field				Garlic field			
		H_0_T	H_0_T_0_	HT	HT_0_	H_0_T	H_0_T_0_	HT	HT_0_	H_0_T	H_0_T_0_	HT	HT_0_	H_0_T	H_0_T_0_	HT	HT_0_
Annual species																	
1. *Acalypha australis* L.	F	8.7	9.0	2.7	–	15.0	8.8	13.8	4.6	–	–	–	–	1.0	1.0	–	–
2. *Amaranthus retroflexus* L.	F	3.8	–	–	–	–	–	–	–	–	–	–	–	–	–	–	–
3. *Calystegia hederacea* Wall	F	–	2.4	–	–	–	–	–	–	–	15.1	–	–	–	–	14.6	8.9
4. *Celosia argentea* L.	F	–	–	–	–	0.3	0.2	–	4.5	–	–	–	–	–	–	–	–
5. *Chenopodium album* L.	F	–	–	–	–	–	–	–	–	–	–	–	–	50.4	54.0	38.9	34.4
6. *Cucumis melo* L. var. agrestis Naud.	F	13.3	14.5	51.2	54.9	–	–	54.7	50.0	–	–	–	–	–	–	–	–
7. *Descurainia sophia* L. Webb ex Prantl	F	–	–	–	–	–	–	–	–	62.5	33.4	75.6	74.2	12.3	9.8	7.8	14.4
8. *Digitaria ciliaris* var*. chrysoblephara*	G	12.2	17.6	22.1	34.4	11.0	2.8	10.3	–	–	–	–	–	–	0.4	–	–
9. *Echinochloa crus-galli* var*. zelayensis* (Kunth) Hitchcock	G	28.9	16.6	6.8	7.2	19.2	35.2	–	–	–	–	–	–	–	–	–	–
10. *Eclipta prostrata* L.	F	3.0	2.0	6.5	3.6	–	–	–	–	–	–	–	–	–	–	–	–
11. *Eleusine indica* L. Gaertn.	G	1.7	13.6	–	–	10.8	18.8	–	–	–	–	–	–	–	–	–	–
12. *Polygonum aviculare* L.	F	–	–	–	–	–	–	–	–	–	–	–	–	3.4	3.9	3.6	0.1
13. *Portulaca oleracea* L.	F	5.2	5.3	1.2		39.0	17.0	15.5	10.7	–	–	–	–	–	–	–	–
14. *Setaria viridis* L. P.Beauv.	G	14.3	9.0	–	–	4.8	14.6	–	–	–	–	–	–	8.7	11.0	–	5.9
Perennial species																	
1. *Cirsium setosum* (Willd.) MB.	F	–	1.8	1.5	–	–	2.6	1.7	11.6	25.0	35.4	16.8	16.4	4.8	4.2	17.6	17.5
2. *Convolvulus arvensis* L.	F	0.3	5.3	–	–	–	–	2.6	6.7	–	–	–	–	2.0	1.3	7.7	6.7
3. *Cyperus iria* L.	G	8.7	4.2	8.1	–	–	–	1.6	2.3	–	–	–	–	–	–	–	–
Annual + Biannual species																	
1. *Capsella bursa-pastoris* L. Med.	F	–	–	–	–	–	–	–	–	12.5	16.2	7.6	2.9	15.0	13.3	10.0	12.2
Annual + Perennial species																	
1. *Humulus scandens* (Lour.) Merr.	F	–	–	–	–	–	0.1	–	–	–	–	–	6.6	2.5	1.2	–	–

**Note:**

H_0_T, tillage without herbicide; H_0_T_0_, without herbicide and tillage; HT, both herbicide and tillage; HT_0_, herbicide without tillage; Plant type: F, Forb; G, Graminoid.

### Weed biomass and density

**Figure 3:**
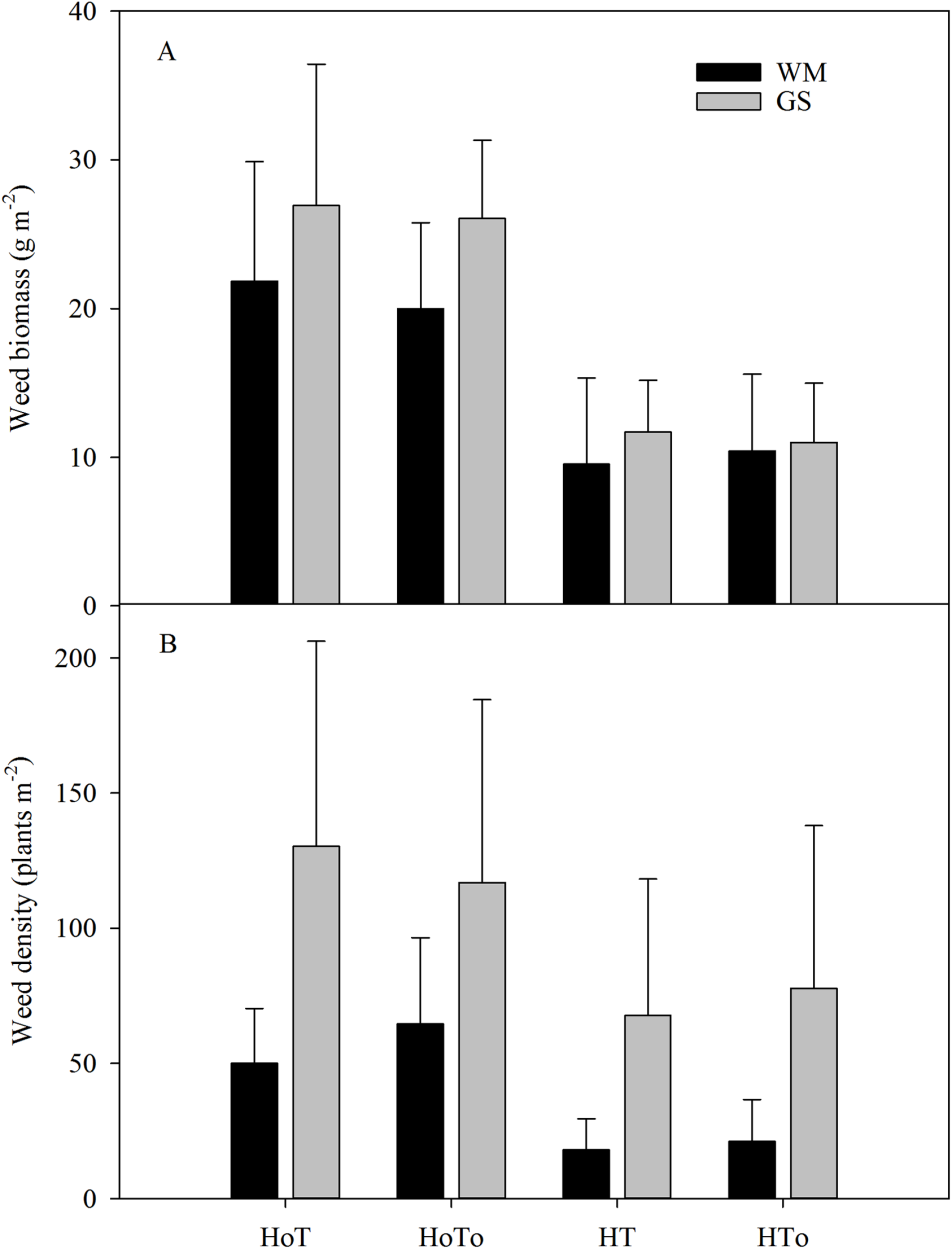
The total weeds biomass (A) and weed density (B) in wheat–maize (WM) and garlic–soybean (GS) rotation systems with different weed and tillage managements. Data of columns are means ± SE. H_0_T, tillage without herbicide; H_0_T_0_, without herbicide and tillage; HT, both herbicide and tillage; HT_0_, herbicide without tillage.

The total weed biomass was much higher in the no-herbicides treatments (H_0_T, H_0_T_0_) than that of herbicides ones (HT, HT_0_) in both rotations. The highest weed biomass appeared in H_0_T treatment. However, the weed biomass in GS was much higher than that of WM under the same treatment. For instance, weed biomass in GS was 18.8% higher than that of WM in H_0_T treatment. Herbicides application led to more than 40% reduction in weed biomass in both rotations (Fig. 3A). Three-way ANOVA test resulted in a significant interaction for only the herbicide factor (*F* = 8.93, *P* < 0.01) (Table S2).

The change of weed density was very similar to weed biomass. Weed density of GS rotation was much higher than that of WM rotation under the same treatment. In H_0_T treatment of GS rotation, the weed density reached to 130 plants · m^2^, which was the maximum density among the four treatments (Fig. 3B). Three-way ANOVA test results showed that a significant interaction was occurred in the rotation factor (*F* = 3.12, *P* < 0.05) (Table S2).

### Size and distribution of seed bank

**Figure 4:**
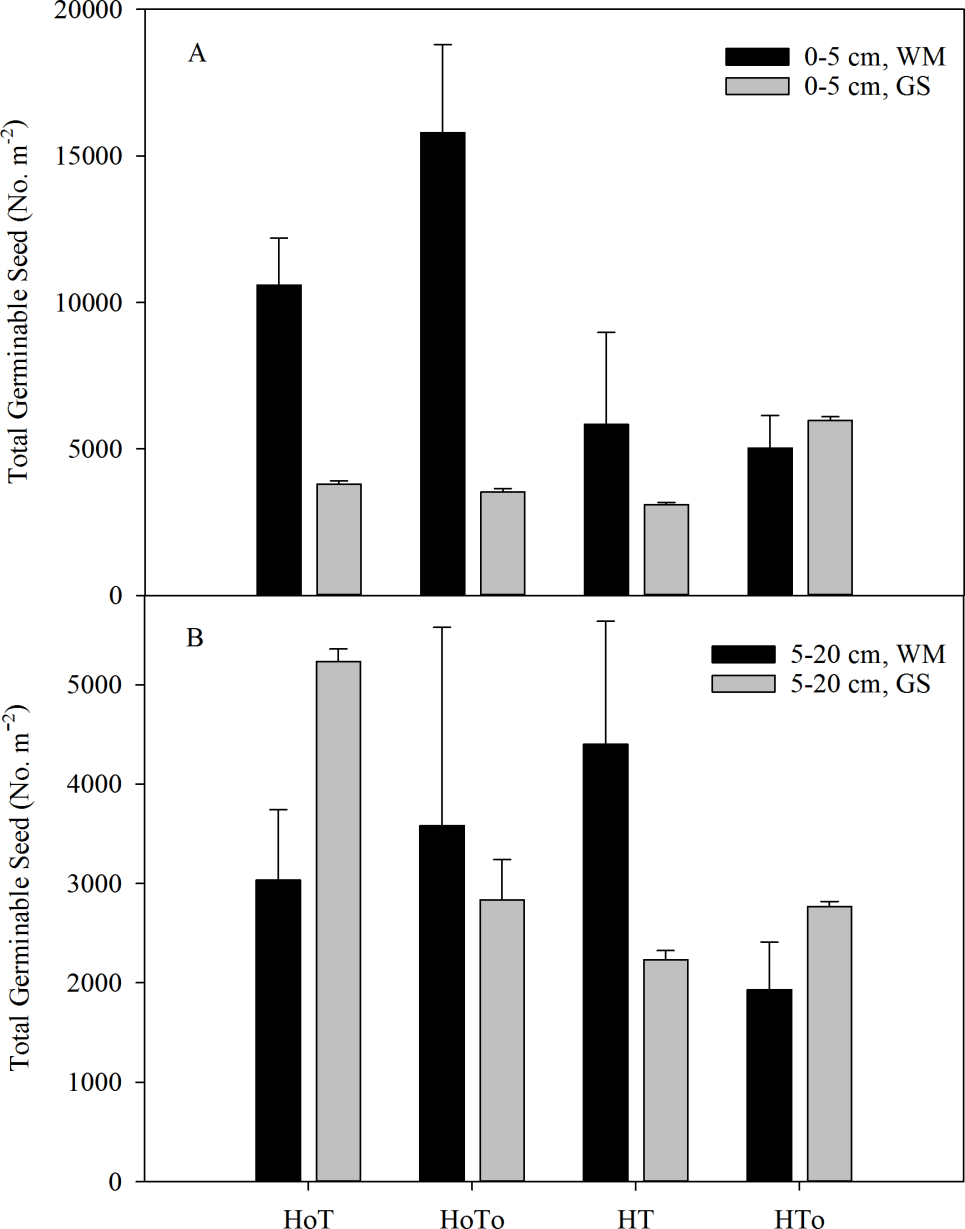
Total germinable seeds in the soil level of 0–5 cm (A) and 5–20 cm (B) in wheat–maize (WM) and garlic–soybean (GS) rotation systems with different weed and tillage managements. Data of columns are means ± SE. H_0_T, tillage without herbicide; H_0_T_0_, without herbicide and tillage; HT, both herbicide and tillage; HT_0_, both herbicide without tillage.

In 0–5 cm soil layer, the total germinable weed seed densities of the four treatments varied from 4,766 to 15,800 No. · m^−2^, with H_0_T_0_ having the highest seed density in the WM rotation; In the GS rotation, seed bank varied from 3,100 to 5,966 No. · m^−2^, with HT_0_ having the highest seed density. The total seed bank in WM was 137% larger than that of GS (Fig. 4A). Three-way ANOVA test results showed a significant interaction in the rotation factor (*F* = 28.78, *P* < 0.001) and herbicide (*F* = 12.59, *P* < 0.01) (Table S3). In 5–20 cm soil layer, the seed bank varied from 1,933 to 4,400 No. · m^−2^ in the WM rotation (Fig. 4B). In GS, the seed bank changed from 2,233 to 5,233 No. · m^−2^, with H_0_T having the largest one. Three-way ANOVA test results showed that there was no significant interaction in the rotation, herbicide and tillage factors in this soil layer (Table S3).

### Chemical, physical and biological properties of the soil

**Figure 5:**
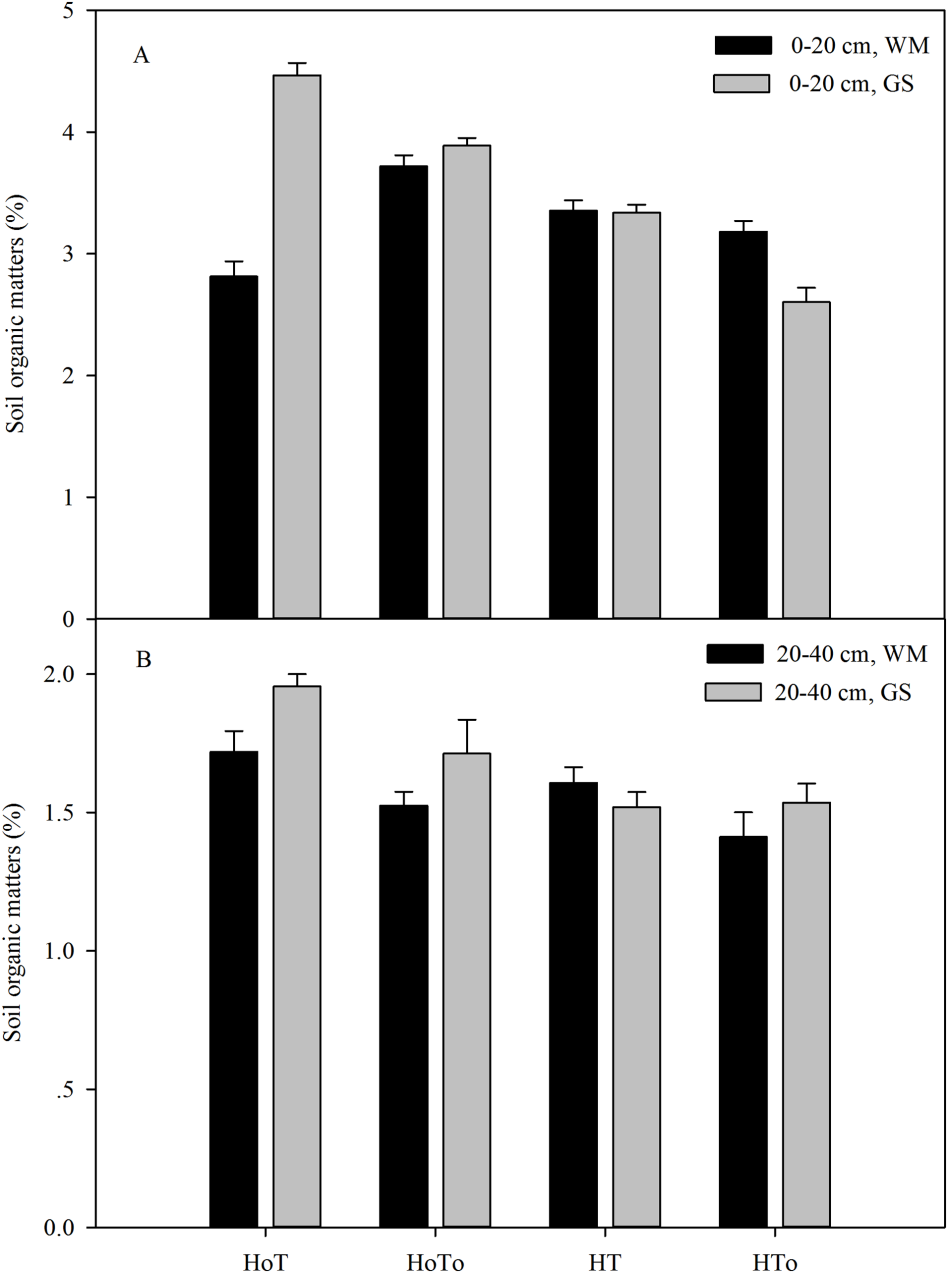
Soil organic matter of the soil level of 0–20 cm (A) and 20–40 cm (B) in wheat–maize (WM) and garlic–soybean (GS) rotation systems with different weed and tillage managements. Data of columns are means ± SE. H_0_T, tillage without herbicide; H_0_T_0_, without herbicide and tillage; HT, both herbicide and tillage; HT_0_, both herbicide without tillage.

In 0–20 cm soil layer, the treatments without herbicide (H_0_T_0_, H_0_T) had higher SOM content in GS than that in WM. H_0_T had the highest SOM in GS, while the highest SOM was noted in H_0_T_0_ in WM (Fig. 5A). Three-way ANOVA test results showed that a significant interaction occurred among the rotation, herbicide and tillage factors (*P* < 0.001) (Table S4). In 20–40 cm soil layer, SOM in GS was generally higher than that in WM; SOM under herbicide-free treatments (H_0_T and H_0_T_0_) was higher than that of the herbicide treatments (HT and HT_0_), the highest SOM appeared in H_0_T treatment, while the lowest SOM was noticed in HT_0_ (Fig. 5B). Three-way ANOVA analysis displayed a significant interaction in the factors of rotation (*F* = 4.81, *P* < 0.01), herbicide (*F* = 16.06, *P* < 0.001) and tillage (*F* = 8.67, *P* < 0.01) (Table S4).

**Figure 6:**
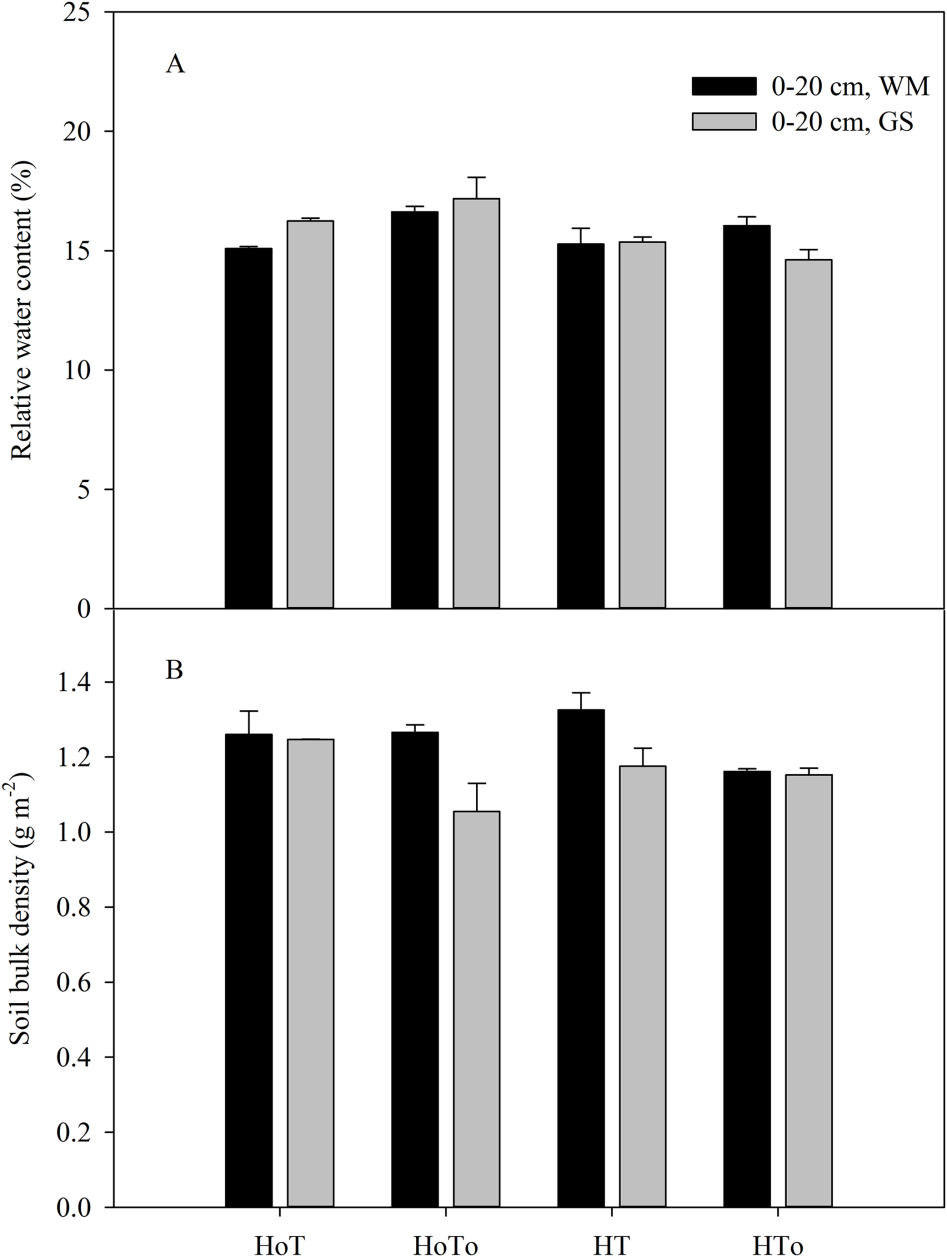
Soil bulk density (A) and relative water content (B) in wheat–maize (WM) and garlic–soybean (GS) rotation systems with different weed and tillage managements. Data of columns are means ± SE. H_0_T, tillage without herbicide; H_0_T_0_, without herbicide and tillage; HT, both herbicide and tillage; HT_0_, both herbicide without tillage.

In WM, the highest relative water content (RWC) (16%) was obtained in H_0_T_0_ treatment, followed by the HT_0_, whereas the lowest value (14%) was in the H_0_T treatment. In GS rotation, the RWC varied from 14% to 17% (Fig. 6A). A significant interaction was noted in factors of herbicide (*F* = 8.76, *P* < 0.01), tillage (*F* = 3.76, *P* < 0.05) (Table S4). Nevertheless, there were no significant differences between the two rotations under the same condition. Soil bulk density is displayed different trend as RWC (Fig. 6B). Three-way ANOVA test results showed a significant interaction in the factors of rotation (*F* = 9.97, *P* < 0.01), tillage (*F* = 9.54, *P* < 0.01), with rotation*herbicide*tillage mixed factors being significantly related (*F* = 8.05, *P* < 0.01) (Table S4).

**Figure 7:**
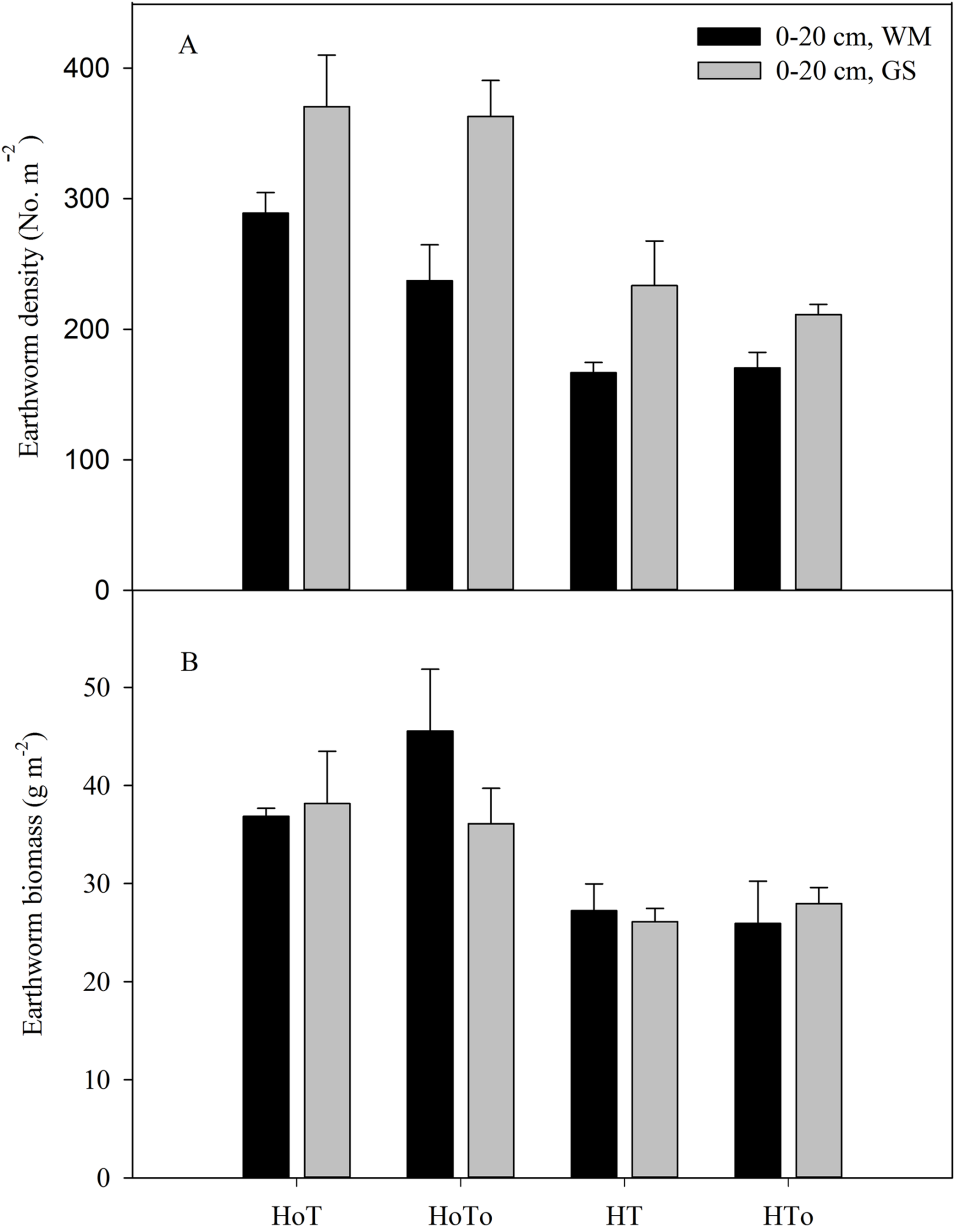
Soil earthworm density (A) and earthworm biomass (B) in wheat–maize (WM) and garlic–soybean (GS) rotation systems with different weed and tillage managements. Data of columns are means ± SE. H_0_T, tillage without herbicide; H_0_T_0_, without herbicide and tillage; HT, both herbicide and tillage; HT_0_, both herbicide without tillage.

The highest earthworm densities of 0–20 cm layer emerged in H_0_T treatment, which were 289 and 370 No. · m^−2^, respectively in WM and GS (Fig. 7A). A significant interaction occurred in rotation (*F* = 31.1, *P* < 0.001) and herbicide (*F* = 71.7, *P* < 0.001) factors (Table S4). The application of herbicide resulted in a great decrease in earthworm density (Fig. 7A). Both rotations presented similar variation tendency, however, GS rotation always had higher earthworm densities than that of WM under the same treatment. Similar to earthworm density, herbicide treatments (HT, HT_0_) had lower earthworm biomass in both rotations (Fig. 7B). A significant interaction was found in the herbicide factor (*F* = 32.67, *P* < 0.001) (Table S4). However, there were no significant differences between WM and GS under the same treatment (Fig. 7B).

### Plant height, leaf area index, and aboveground biomass

For plant height, a significant interaction occurred in the crop species factor (*F* = 1088.7, *P* < 0.001) and the herbicide factor (*F* = 13.01, *P* < 0.001), respectively (Table S5). For LAI, there was significant interaction in the crop species (*F* = 175.45, *P* < 0.001), tillage (*F* = 17.49, *P* < 0.001) and crop species*tillage mixed factors (*F* = 7.48, *P* < 0.001) (Table S5). Herbicide treatments (HT, HT_0_) had higher crop LAI than that of no-herbicide treatments (H_0_T, H_0_T_0_) in summer maize field. LAI demonstrated similar change trend in soybean field. Furthermore, LAI was greatly influenced by tillage management. Treatments with primary plowing displayed higher LAI than that of treatments without plowing under the same condition (Table 3). Crops species was the only factor affecting aboveground biomass by the ANOVA analysis (*F* = 609.8, *P* < 0.001) (Table S5). In WM rotation, herbicide and tillage had no significant effects on the aboveground biomass of the two crops. However, in GS rotation, the aboveground biomass was much higher in the herbicide-free treatments than that of herbicide ones, especially for garlic crop. Nevertheless, the aboveground biomass of soybean showed an opposite trend (Table 3).

**Table 3:** Plant height, leaf area index, and aboveground biomass of the different crops at the harvesting stage of 2015–2016.

	Winter wheat			Summer maize			Garlic			Soybean		
	H	LAI	ABM	H	LAI	ABM	H	LAI	ABM	H	LAI	ABM
**H_0_T**	76.8 ± 0.7	5.5 ± 0.1	12.3 ± 1.2	235.5 ± 1.6	4.3 ± 0.2	17.0 ± 0.5	74.2 ± 1.6	2.1 ± 0.1	1.8 ± 0.1	91.8 ± 5.2	4.2 ± 0.1	6.4 ± 1.1
**H_0_T_0_**	77.7 ± 0.9	5.0 ± 0.2	11.6 ± 0.5	228.6 ± 16.3	3.9 ± 0.1	16.7 ± 0.9	71.7 ± 0.8	2.1 ± 0.2	1.8 ± 0.1	75.8 ± 4.8	3.7 ± 0.1	7.2 ± 6.0
**HT**	79.3 ± 2.0	5.4 ± 0.2	11.7 ± 0.3	245.8 ± 2.7	4.6 ± 0.1	18.6 ± 0.6	75.2 ± 1.9	2.2 ± 0.1	1.4 ± 0.1	101.5 ± 1.0	4.9 ± 0.1	8.6 ± 1.1
**HT_0_**	77.3 ± 0.9	5.3 ± 0.2	11.2 ± 0.4	241.3 ± 2.8	4.6 ± 0.1	17.3 ± 0.7	77.7 ± 1.4	2.2 ± 0.3	1.7 ± 0.2	102.3 ± 3.3	3.7 ± 0.3	9.4 ± 3.5

**Note:**

H, plant height (cm); LAI, means leaf area index; ABM, aboveground biomass (t·ha^−1^); H_0_T, tillage without herbicide; H_0_T_0_, without herbicide and tillage; HT, both herbicide and tillage; HT_0_, herbicide without tillage.

### Yield and economic performances

In WM, the averaged total yield of wheat and maize was the highest in HT treatment, followed by H_0_T, HT_0_, and H_0_T_0_. In GS rotation, the highest yield appeared in H_0_T, followed by H_0_T_0_, HT_0_, HT treatments (Table 4). ANOVA results showed a significant interaction in factors of rotation (*F* = 36.2, *P* < 0.001), herbicide (*F* = 4.3, *P* < 0.01), tillage (*F* = 11.4, *P* < 0.01) and rotation*herbicide mixed factor (*F* = 6.0, *P* < 0.01) (Table S6).

**Table 4:** Comparisons of yield, output, input and the net income of wheat–maize (WM) and garlic–soybean (GS) rotations with different weed and tillage managements.

Items	Years	Rotations	H_0_T	H_0_T_0_	HT	HT_0_
**Yield** (t·ha^−1^)	2015	WM	13.5 ± 0.6	13.5 ± 1.2	15.1 ± 0.2	14.3 ± 0.2
		GS	12.4 ± 0.4	11 ± 0.6	9.9 ± 0.6	10.6 ± 0.4
	2016	WM	16.4 ± 0.4	15.1 ± 0.2	16.4 ± 0.2	14.8 ± 0.3
		GS	14.8 ± 0.7	14.8 ± 0.4	12.0 ± 0.8	11.3 ± 0.8
**Output** (US$·ha^−1^)	2015	WM	12378 ± 583.6	12398 ± 1097.0	4668.5 ± 54.1	4421.2 ± 75.4
		GS	20118.5 ± 722.0	17685 ± 935.8	7073.9 ± 510.0	7476.2 ± 264.6
	2016	WM	15007.3 ± 369.1	13887.4 ± 172.0	5080.4 ± 53.0	4592.9 ± 96.0
		GS	22712.3 ± 1020.9	22760.7 ± 642.8	7861.7 ± 493.6	7433.8 ± 551.2
**Net income** (US$·ha^−1^)	2015	WM	9443.8 ± 583.6	9560.6 ± 1097.0	1677.9 ± 54.1	1527.4 ± 75.4
		GS	17083.1 ± 722.0	14746.4 ± 935.8	3885.9 ± 510.0	4385 ± 264.6
	2016	WM	12083.1 ± 369.1	11050.1 ± 172.0	2089.8 ± 53.0	1699.2 ± 96.3
		GS	19676.9 ± 1020.9	19822.1 ± 642.8	4673.6 ± 493.6	4342.6 ± 551.2

**Note:**

H_0_T, tillage without herbicide; H_0_T_0_, without herbicide and tillage; HT, both herbicide and tillage; HT_0_, herbicide without tillage.

Economical inputs included the cost of seed, fertilizer, irrigation, tillage, herbicides, and labor. As shown in Table S1, the application of herbicides and tillage increased the inputs of production. Therefore, inputs in the herbicides or tillage treatments were higher than that of herbicides or tillage free ones in both rotations. Meanwhile, the inputs of GS were much higher than that of WM under the same condition.

For WM, the outputs in herbicides-free treatments were about three to four times of that in herbicides ones. GS rotation had higher output than WM under the same conditions. Net income appeared a similar trend with the output. Net income of herbicide-free treatments was about five to six times of herbicides ones in WM. For GS, net incomes of herbicide-free treatments (H_0_T, H_0_T_0_) were about 186% and 179% higher than the herbicide treatments (HT, HT_0_). Moreover, GS had 69% higher net income than WM for their organic products (Table 4). According to the ANOVA analysis, rotation (*F* = 158.8, *P* < 0.001), herbicide (*F* = 1486.2, *P* < 0.001), tillage (*F* = 2.3, *P* < 0.05) and year (*F* = 49.5, *P* < 0.001) were the factors affecting net income (Table S6).

## Discussion

Many factors can affect weed population and composition, such as rainfall, air temperature, sunlight (Yunusa et al., 1993; Kleijn & van der Voort, 1997; Yin, Cai & Zhong, 2005), soil moisture, soil temperature, and nutrition (Tang et al., 2014). Soil seed bank contributes largely to the weed community (Bàrberi & Cascio, 2001; Albrecht, 2005). In order to control weed wisely, the weed community should be managed from the very beginning by depressing their vegetation growth. Application of herbicide and artificial weeding are both popular methods. In our study, three-way ANOVA test results showed herbicide was an important factor that affected soil seed bank, SOM, soil water content, earthworm biomass and density, plant height and yield (Tables S3–S6). Although herbicides could temporarily control weeds, the weeds still germinate in the later stage (Table 2), and long term application of chemical herbicides has caused serious environmental and food pollutions worldwide (Liu et al., 2016; Meng et al., 2016). Actually, weeds have ecological function, such as maintaining soil nutrient cycle, soil moisture and providing habitats for insect enemies (Altieri, 1999). In our study, we found that without herbicides the weed biomass and density were higher than that with herbicides (Fig. 3), but the SOM (Fig. 5), soil water content (Fig. 6A), earthworm density, and biomass (Fig. 7), especially the yields were also higher (Table 4). Therefore, moderate weeds in the field might be good for the soil to maintain the nutrient and crops to grow.

Weed community is also influenced by other factors, such as crop covering, architectural structure of canopies, allelopathy, crop rotation (Liebman & Dyck, 1993; Jabran et al., 2015), and fertilization (Yunusa et al., 1993; Kleijn & van der Voort, 1997; Yin, Cai & Zhong, 2005, 2006). Although the total weed species numbers did not change largely, it was observed that crop rotation did alter weed biomass and weed density. Both weed biomass and density in WM rotation were lower than that in GS rotation, especially in cases of none herbicide usages (Fig. 3). Light is one of the most important factors for weed growth and competition (Yin, Cai & Zhong, 2005). Crops might be manipulated to increase shading of weeds by the crop canopy, to cease growth of weeds so as to increase crop yield (Teasdale & Mohler, 1993; Chauhan, Gill & Preston, 2006; Borger, Hashem & Pathan, 2010). Crop rotation could change the shading by changing the crop to alter the dominant weed species and seed bank, therefore, improving weed management strategies may be possible through rotational schemes.

None tillage system could increase the annual weed abundance and reduce the variety of weeds, with the richness and consistency in composition by crops being disturbed year by year (Menalled, Gross & Hammond, 2001). The reduced tillage such as mouldboard ploughing could effectively decrease annual species (Sans et al., 2011), however increase perennial weeds after several years (Gruber & Claupein, 2009). Conservation tillage, especially non-tillage could strongly affect the seed germination environment due to changes in the temperature and moisture of the upper soil layers and the retention of crop residues on the soil surface (Froud-Williams, 1988). From this study, soil moisture, the number and density of earthworms in the non-tillage treatment were found to be higher than that in the tillage treatment (Figs. 6B and 7), that might be because there was no disturbance to the soil. Without herbicide and no-tillage (H_0_T_0_) was found increasing soil germinable seed bank and weed density (Figs. 3 and 4). Part of the reason might be that without herbicide and tillage, soil moisture was fit to the weed to grow and increased the annual weeds.

Organic management contributes to enhancing ecological profits by increasing soil fertility and earthworms, microbiological amounts, and controlling of pests (Liu et al., 2016; Meng et al., 2016). Based on the economic analysis, we found that organic management of weeds was more expensive than chemical one, however, organic method was more environmentally friendly. For farmers, if their products were sold with a higher price, for instance more than three to five times higher, they were happy to take the organic approach. In this study, crop yields were not found to be significantly different among different treatments, especially for the herbicide and non-herbicide treatments. According to organic standards, grains or vegetables if polluted by herbicides can only be sold in accordance with ordinary products. When sold at organic prices, the economic benefits of WM and GS increased by five and nine times, respectively. From the economic point of view, using organic methods to control weeds and care for cropland are feasible and practicable. In addition, under organic condition, GS rotation had 69% higher income than WM. So we might believe that planting organic garlic and soybean could not only nurse the cropland but also yield higher economic output.

## Conclusion

In conclusion, soil moisture, the number and density of earthworms in the non-tillage treatment were found to be higher than that in the tillage treatment. Soil seed bank was bigger in the organic field, without herbicide and no-tillage was found increasing soil germinable seed bank and weed density, but herbicides only temperately controlled weeds which further caused soil and food pollution. Organic weed control method could increase SOM, soil moisture and earthworms which are beneficial to the soil productivity. Crop rotation was tested to be successful and environmentally friendly in weed control.

## Supplemental Information

10.7717/peerj.4799/supp-1 Supplemental Information 1 Table S1. Detailed lists of crop inputs for organic and non-organic weed managements in different crop rotations. “*” represents wheat and maize crop rotation system management. “**” represents garlic and soybean crop rotation system management..

10.7717/peerj.4799/supp-2 Supplemental Information 2 Table S2. Multivariate analysis of variance by three way ANOVA of the weed biomass (WB; n = 8) and weed density (WD; n = 8). The categorical factors are rotation, herbicide and tillage. Presented are the F-values with the level of significance; *P<0.05, **P<0.01, ***P<0.001, ^n.s.^-no significant.

10.7717/peerj.4799/supp-3 Supplemental Information 3 Table S3. Multivariate analysis of variance by three way ANOVA of the soil seed bank; total garminable seed (n = 3) with two level depth (0–5 cm and 5–20cm). The categorical factors are rotation, herbicide and tillage. Presented are the F-values with the level of significance; *P<0.05, **P<0.01, ***P<0.001, ^n.s.^-no significant.

10.7717/peerj.4799/supp-4 Supplemental Information 4 Table S4. Multivariate analysis of variance by three way ANOVA of the soil chemical properties including soil organic matter (SOM; n = 3) with two level 0–20 cm and 20–40 cm. Soil physical properties including soil bulk density (SBD; n = 3) and relative water content (RWC; n = 3) with the 0–20 cm depth and soil biological properties including soil earthworm density (SED; n = 3) and biomass (SEB; n = 3) with the 30 cm (length) × 30 cm (width) × 20 cm (depth).
The categorical factors are rotation, herbicide and tillage. Presented are the F-values with the level of significance; *P<0.05, **P<0.01, ***P<0.001, ^n.s.^-no significant.

10.7717/peerj.4799/supp-5 Supplemental Information 5 Table S5. Multivariate analysis of variance by three-way ANOVA of the crop morphological parameters including plant height (H; n = 12), leaf area index (LAI; n = 12), and crop productivity parameter including aboveground biomass (ABM: n = 12). The categorical factors are crop species (including the winter wheat, summer maize, garlic and soybean), herbicide and tillage. Presented are the F-values with the level of significance; *P<0.05, **P<0.01, ***P<0.001, ^n.s.^-no significant.

10.7717/peerj.4799/supp-6 Supplemental Information 6 Table S6. Multivariate analysis of variance by four-way ANOVA of the economical details including; crop yield (n = 12), output (n = 12) and net income (n = 12). The categorical factors are year, rotation, herbicide and tillage. Presented are the F-values with the level of significance; *P <0.05, **P <0.01, ***P<0.001, ^n.s.^-no significant.

## Additional Information and Declarations

### Field Study Permissions

The following information was supplied relating to field study approvals (i.e., approving body and any reference numbers): Field experiments were approved by the Plant Eco-physiological Research Group in the Institute of Botany, Chinese Academy of Sciences (project number: 201405.5).

### Data Availability

The following information was supplied regarding data availability: The raw data are provided in the Supplemental Files.
