# Supplementary material for: Comparisons of weed community, soil health and economic performance between wheat-maize and garlic-soybean rotation systems under different weed managements
Source: PeerJ. 2018 May 30;6:e4799. doi: 10.7717/peerj.4799 (PMC5984582; doi:10.7717/peerj.4799)
Supplement: Supplemental Information 1 — “*” represents wheat and maize crop rotation system management. “**” represents garlic and soybean crop rotation system management.. [file peerj-06-4799-s001.docx]

Table S1 Detailed lists of crop inputs for organic and non-organic weed managements in different crop plots.

| Details of Crop Inputs, amount ($ ha^-1^) | | | | | | |
| --- | --- | --- | --- | --- | --- | --- |
| Content | Details | Price Schedules | H_0_T | H_0_T_0_ | HT | HT_0_ |
| Machine | Transportation organic compost “*”“**”  Transportation biogas slurry “*”“**”  Corn seeder “*”  Wheat seeder “*”  Combine harvester for wheat “*”  Combine harvester for corn “*”  Rotary tillage fare before winter crop seeding“*” “**”  Plough for summer crops “*”“**”  Solar energy light-traps depreciation costs “*”“**” | 3.23$ CAR^-1^ *22.5 CAR ha^-1^  12.9 $ CAR^-1^, 9 CAR ha^-1^  69.2 $ ha^-1^  69.2 $ ha^-1^  184.6 $ ha^-1^  184.6 $ ha ^-1^  Mechanical rotary tillage, 121.05 $ ha^-1^  96.84 $ ha^-1^  322.8 $ light^-1^, 1 light ha^-1^,  5 years for 1 light | 72,6  116,2  69,2  69.2  184,6  184,6  121,05  96,84  64,6 | 72,6  116,2  69,2  69,2  184,6  184,6  121,05  0,0  64,6 | 72,6  116,2  69,2  69,2  184,6  184,6  121,05  96,84  0,0 | 72,6  116,2  69,2  69,2  184,6  184,6  121,05  0,0  0,0 |
| Irrigation | Requirements for irrigation to government “*” “**” | 217.89 $ ha^-1^ | 436,0 | 436,0 | 436,0 | 436,0 |
| Labor | Composting “*”“**”  Manual weeding “*”“**”  Fertilizers application “*”“**”  Spray herbicide “*”“**”  Spray insecticide “*”“**”  Drainage “*”“**”  **Sowing**  Soybean “**”  Garlic “**”  **Harvesting**  Soybean “**”  Garlic“**”  **Irrigation**  Corn “*”  Soybean“**”  Wheat “*”  Garlic“**” | 2 labor ha^-1^, once a year  15 labor ha^-1^, four times a year  15 labor ha^-1^, once a year  4 labor ha^-1^ , four times a year  4 labor ha^-1^, twice a year  4 labor ha^-1^, once a season  15 labor ha^-1^, once a season  15 labor ha^-1^, once a season  18 labor ha^-1^, once a year  18 labor ha^-1^, once a year  9 labor ha ^-1^ , once a quarter  9 labor ha ^-1^, once a quarter  18 labor ha ^-1^, twice a season  27 labor ha ^-1^ , thrice a season | 19,4  145,2  145,3  0,0  0,0  43,6  174,3  174,3  173,3  173,3  23,1  23,1  46,2  69,2 | 19,4  145,2  145,3  0,0  0,0  43,6  174,3  174,3  173,3  173,3  23,1  23,1  46,2  69,2 | 19,4  0,0  145,3  72,6  43,6  43,6  174,3  174,3  173,3  173,3  23,1  23,1  46,2  69,2 | 19,4  0,0  145,3  72,6  43,6  43,6  174,3  174,3  173,3  173,3  23,1  23,1  46,2  69,2 |
| Fertilizer | Organic fertilizers “*”“**”  Biogas slurry “*”“**” | 7.5 t/ ha^-1^, 4.84$ m^-1^  60 cube ha^-^, 2.98$ m^-3^ | 363,2  179 | 363,2  179 | 363,2  179 | 363,2  179 |
| Herbicide | Paraquat for wheat and garlic “*”“**”  Acetochlor for maize and soybean “*”“**” | 15 bottle ha^-1^, 1.6 $ bottle^-1^,  24.21 $ ha^-1^ | 0,0 | 0,0 | 24,2 | 24,2 |
| Pesticide | Phoxim for wheat and garlic “*”“**”  Imidacloprid for corn and soybean “*”“**” | 15 bottle ha^-1^, 1.6 $ bottle^-1^,  24.21 $ ha^-1^ | 0,0 | 0,0 | 24,2 | 24,2 |
| Seeds | Corn “*”  Wheat “*”  Soybean“**”  Garlic“**” | 0.48 $ kg^-1^, 225 kg ha^-1^  3.23 $ kg^-1^, 30 kg ha^-1^  0.69 $ kg^-1^, 45 kg ha^-1^  1.19 $ kg^-1^, 1500 kg ha^-1^ | 109,0  96,8  30,9  1785,0 | 109,0  96,8  30,9  1785,0 | 109,0  96,8  30,9  1785,0 | 109,0  96,8  30,9  1785,0 |
| Sum | Wheat and maize rotation system (WM) |  | 2585,69 | 2488,85 | 2605,09 | 2508,25 |
|  | Garlic and soybean rotation system (GS) |  | 4407,39 | 4309,55 | 4425,79 | 4328,95 |

“*” represents wheat and maize crop rotation system management.

“**” represents garlic and soybean crop rotation system management.
