# Supplementary material for: Comparisons of weed community, soil health and economic performance between wheat-maize and garlic-soybean rotation systems under different weed managements
Source: PeerJ. 2018 May 30;6:e4799. doi: 10.7717/peerj.4799 (PMC5984582; doi:10.7717/peerj.4799)
Supplement: Supplemental Information 2 — The categorical factors are rotation, herbicide and tillage. Presented are the F-values with the level of significance; *P<0.05, **P<0.01, ***P<0.001, n.s.-no significant. [file peerj-06-4799-s002.docx]

Table S2 Multivariate analysis of variance by three way ANOVA of the weed biomass (WB; n = 8) and weed density (WD; n = 8)

| Source of variables | df | F-value | |
| --- | --- | --- | --- |
|  |  | WB | WD |
| Rotation | 1 | 0.63^n.s.^ | 3.12* |
| Herbicide | 1 | 8.93** | 1.72^n.s.^ |
| Tillage | 1 | 0.02^n.s^. | 0.01^n.s.^ |
| Rotation*Herbicide | 1 | 0.23^n.s.^ | 0.04^n.s.^ |
| Rotation*Tillage | 1 | 0.01^n.s.^ | 0.02^n.s.^ |
| Herbicide*Tillage | 1 | 0.03^n.s.^ | 0.01^n.s.^ |
| Rotation*Herbicide*Tillage | 1 | 0.02^n.s.^ | 0.07^n.s.^ |

The categorical factors are rotation, herbicide and tillage. Presented are the F-values with the level of significance; **P*<0.05, ***P*<0.01, ****P*<0.001, ^n.s.^-no significant.
