# Supplementary material for: Comparisons of weed community, soil health and economic performance between wheat-maize and garlic-soybean rotation systems under different weed managements
Source: PeerJ. 2018 May 30;6:e4799. doi: 10.7717/peerj.4799 (PMC5984582; doi:10.7717/peerj.4799)
Supplement: Supplemental Information 3 — The categorical factors are rotation, herbicide and tillage. Presented are the F-values with the level of significance; *P<0.05, **P<0.01, ***P<0.001, n.s.-no significant. [file peerj-06-4799-s003.docx]

Table S3 Multivariate analysis of variance by three way ANOVA of the soil seed bank; total garminable seed (n=3) with two level depth (0-5 cm and 5-20cm)

| Sources | df | F-value | |
| --- | --- | --- | --- |
|  |  | 0-5 cm | 5-20 cm |
| Rotation | 1 | 28.78*** | 0.02^n.s.^ |
| Herbicide | 1 | 12.59** | 1.81^n.s.^ |
| Tillage | 1 | 3.24^n.s.^ | 2.84^n.s.^ |
| Rotation*Herbicide | 1 | 19.70*** | 1.59^n.s.^ |
| Rotation*Tillage | 1 | 0.21^n.s.^ | 0.01^n.s.^ |
| Herbicide*Tillage | 1 | 0.54^n.s.^ | 0.002^n.s.^ |
| Rotation*Herbicide*Tillage | 1 | 5.51* | 5.99^n.s.^ |

The categorical factors are rotation, herbicide and tillage. Presented are the F-values with the level of significance; **P*<0.05, ***P*<0.01, ****P*<0.001, ^n.s.^-no significant.
