# Supplementary material for: Comparisons of weed community, soil health and economic performance between wheat-maize and garlic-soybean rotation systems under different weed managements
Source: PeerJ. 2018 May 30;6:e4799. doi: 10.7717/peerj.4799 (PMC5984582; doi:10.7717/peerj.4799)
Supplement: Supplemental Information 4 — Soil physical properties including soil bulk density (SBD; n = 3) and relative water content (RWC; n = 3) with the 0–20 cm depth and soil biological properties including soil earthworm density (SED; n = 3) and biomass (SEB; n = 3) with the 30 cm (length) × 30 cm (width) × 20 cm (depth). The categorical factors are rotation, herbicide and tillage. Presented are the F-values with the level of significance; *P<0.05, **P<0.01, ***P<0.001, n.s.-no significant. [file peerj-06-4799-s004.docx]

Table S4 Multivariate analysis of variance by three way ANOVA of the soil chemical properties including soil organic matter (SOM; n=3) with two level 0-20 cm and 20-40 cm; soil physical properties including soil bulk density (SBD; n=3) and relative water content (RWC; n=3) with the 0-20 cm depth and soil biological properties including soil earthworm density (SED; n=3) and biomass (SEB; n=3) with the 30 cm (length) × 30 cm (width) × 20 cm (depth)

| Sources | df | F-value | | | | | | | | | |  |
| --- | --- | --- | --- | --- | --- | --- | --- | --- | --- | --- | --- | --- |
|  |  | Physical Properties | |  | Biological Properties | |  | | | Chemical Properties | | |
|  |  | SBD | RWC |  | SEB | SED |  | | SOM,  0-20 cm | | SOM,  20-40 cm | |
| Rotation | 1 | 9.97** | 0.08^n.s.^ |  | 0.70^n.s.^ | 31.1*** |  | 21.0*** | | | 4.81** | |
| Herbicide | 1 | 0.01^n.s.^ | 8.76** |  | 32.67*** | 71.7*** |  | 81.3*** | | | 16.06*** | |
| Tillage | 1 | 9.54** | 3.76* |  | 0.70^n.s.^ | 1.90^n.s.^ |  | 4.7** | | | 8.67** | |
| Rotation*Herbicide | 1 | 0.25^n.s.^ | 5.57* |  | 1.1^n.s.^ | 3.14* |  | 81.9*** | | | 3.41* | |
| Rotation*Tillage | 1 | 0.20^n.s.^ | 2.69^n.s.^ |  | 0.77^n.s.^ | 0.11^n.s.^ |  | 58.6*** | | | 0.61^n.s.^ | |
| Herbicide*Tillage | 1 | 0.004^n.s.^ | 3.60* |  | 0.50^n.s.^ | 0.52^n.s.^ |  | 21.5*** | | | 1.52^n.s.^ | |
| Rotation*Herbicide*Tillage | 1 | 8.05** | 0.52^n.s.^ |  | 2.58^n.s.^ | 1.56^n.s.^ |  | 11.9** | | | 1.55^n.s.^ | |

The categorical factors are rotation, herbicide and tillage. Presented are the F-values with the level of significance; **P*<0.05, ***P*<0.01, ****P*<0.001, ^n.s.^-no significant.
