# Supplementary material for: Comparisons of weed community, soil health and economic performance between wheat-maize and garlic-soybean rotation systems under different weed managements
Source: PeerJ. 2018 May 30;6:e4799. doi: 10.7717/peerj.4799 (PMC5984582; doi:10.7717/peerj.4799)
Supplement: Supplemental Information 5 — The categorical factors are crop species (including the winter wheat, summer maize, garlic and soybean), herbicide and tillage. Presented are the F-values with the level of significance; *P<0.05, **P<0.01, ***P<0.001, n.s.-no significant. [file peerj-06-4799-s005.docx]

Table S5 Multivariate analysis of variance by three-way ANOVA of the crop morphological parameters including plant height (H; n=12), leaf area index (LAI; n=12), and crop productivity parameter including aboveground biomass (ABM: n=12)

| Sources | df | F-value | | | |
| --- | --- | --- | --- | --- | --- |
|  |  | Morphological parameters | |  | Productive parameter |
|  |  | H | LAI |  | ABM |
| Crop species | 3 | 1088.7*** | 175.45*** |  | 609.80** |
| Herbicide | 1 | 13.01*** | 4.56^n.s.^ |  | 5.43^n.s.^ |
| Tillage | 1 | 2.09^n.s.^ | 17.49*** |  | 0.15^n.s.^ |
| Crop species *Herbicide | 3 | 2.68^n.s.^ | 0.74^n.s.^ |  | 5.65^n.s.^ |
| Crop species *Tillage | 3 | 0.61^n.s.^ | 7.48*** |  | 1.98^n.s.^ |
| Herbicide*Tillage | 1 | 1.26^n.s.^ | 0.003^n.s.^ |  | 0.07^n.s.^ |
| Crop species *Herbicide*Tillage | 3 | 0.76^n.s.^ | 0.84^n.s.^ |  | 0.37^n.s.^ |

The categorical factors are crop species (including the winter wheat, summer maize, garlic and soybean), herbicide and tillage. Presented are the F-values with the level of significance; **P*<0.05, ***P*<0.01, ****P*<0.001, ^n.s.^-no significant.
