# Supplementary material for: Comparisons of weed community, soil health and economic performance between wheat-maize and garlic-soybean rotation systems under different weed managements
Source: PeerJ. 2018 May 30;6:e4799. doi: 10.7717/peerj.4799 (PMC5984582; doi:10.7717/peerj.4799)
Supplement: Supplemental Information 6 — The categorical factors are year, rotation, herbicide and tillage. Presented are the F-values with the level of significance; *P <0.05, **P <0.01, ***P<0.001, n.s.-no significant. [file peerj-06-4799-s006.docx]

Table S6 Multivariate analysis of variance by four-way ANOVA of the economical details including; crop yield (n=12), output (n=12) and net income (n=12)

| Sources | df | F value | | |
| --- | --- | --- | --- | --- |
|  |  | Yield | Output | Net income |
| Rotation | 1 | 36.2*** | 354.8*** | 158.8*** |
| Herbicide | 1 | 4.3** | 1481.2*** | 1486.2*** |
| Tillage | 1 | 11.4** | 3.4* | 2.3* |
| Rotation * Herbicide | 1 | 6.0** | 74.1*** | 74.1*** |
| Rotation * Tillage | 1 | 3.6* | 0.1^n.s.^ | 0.1^n.s.^ |
| Herbicide * Tillage | 1 | 1.2^n.s.^ | 1.3^n.s.^ | 1.3 ^n.s.^ |
| Rotation * Herbicide * Tillage | 1 | 0.2^n.s.^ | 0.7^n.s.^ | 0.7^n.s.^ |
| Year | 1 | 0^n.s.^ | 49.5*** | 49.5*** |
| Year * Rotation | 1 | 0^n.s.^ | 9.1** | 9.1** |
| Year * Herbicide | 1 | 0^n.s.^ | 26.6*** | 26.6*** |
| Year * Tillage | 1 | 0^n.s.^ | 0^n.s.^ | 0^n.s.^ |
| Year * Rotation * Herbicide | 1 | 0^n.s.^ | 4.6* | 4.6* |
| Year * Rotation * Tillage | 1 | 0^n.s.^ | 1.6^n.s.^ | 1.6^n.s.^ |
| Year * Herbicide * Tillage | 1 | 0^n.s.^ | 1.1^n.s.^ | 1.1^n.s.^ |
| Year * Rotation * Herbicide * Tillage | 1 | 0^n.s.^ | 3.3* | 3.3* |

The categorical factors are year, rotation, herbicide and tillage. Presented are the F-values with the level of significance; * *P* <0.05, ** *P* <0.01, ****P*<0.001, ^n.s.^-no significant.
